# A light‐triggered Time‐Resolved X‐ray Solution Scattering (TR‐XSS) workflow with application to protein conformational dynamics

**DOI:** 10.1002/2211-5463.70200

**Published:** 2026-02-11

**Authors:** Fatemeh Sabzian‐Molaei, Fredrik Orädd, Konstantinos Magkakis, Magnus Andersson

**Affiliations:** ^1^ Department of Chemistry Umeå University Sweden

**Keywords:** kinetic modeling, protein conformational dynamics, structural refinement, time‐resolved X‐ray solution scattering (TR‐XSS)

## Abstract

Time‐resolved X‐ray solution scattering (TR‐XSS) is a synchrotron‐based methodology that enables real‐time structural characterization under near‐native conditions to provide insight into dynamic and transient structural changes inaccessible to static high‐resolution methods such as cryo‐electron microscopy (cryo‐EM) or X‐ray crystallography. Here, we present a workflow for light‐triggered TR‐XSS experiments that spans data collection, data processing, kinetic analysis, and structural refinement, with accompanying Python scripts. A calcium‐transporting P‐type ATPase membrane protein (LMCA1) is used as an illustrative example, but the protocol is broadly applicable to diverse protein systems. This workflow offers a practical framework for collecting TR‐XSS synchrotron data and subsequent data analysis and interpretation.

## Graphical abstract

Time‐resolved X‐ray solution scattering captures how proteins change shape in real time under near‐native conditions. This article presents a practical workflow for light‐triggered TR‐XSS experiments, from data collection to structural refinement. Using a calcium‐transporting membrane protein as an example, the approach can be broadly applied to study dynamic protein processes.

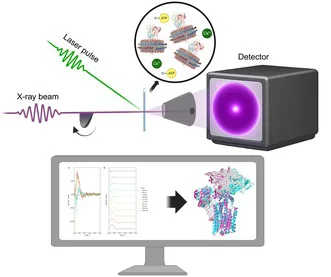

**Abbreviations**
AI: artificial intelligence
ATP: adenosine triphosphate
CAS: Chemical Abstracts Service
CHARMM‐GUI: CHARMM graphical user interface
cryo‐EM: cryo‐electron microscopy
DDM: *n*‐dodecyl‐β‐D‐maltoside
ESRF: European Synchrotron Radiation Facility
FTIR: Fourier‐transform infrared spectroscopy
HPC2N: High‐Performance Computing Center North
IR: infrared
LMCA1: *Listeria monocytogenes* calcium‐transporting P‐type ATPase
LSV: left singular vector
MD: molecular dynamics
MSA: multiple sequence alignment
NMA: normal mode analysis
NPE: nitro‐phenyl‐ethyl
PCA: principal component analysis
PLUMED: plugin for molecular dynamics
POPG: 1‐Palmitoyl‐2‐oleoyl‐sn‐glycero‐3‐(phospho‐rac‐(1‐glycerol))
RMSD: root‐mean‐square deviation
RSV: right singular vector
SAXS: small‐angle X‐ray scattering
SVD: singular value decomposition
TR‐XSS: time‐resolved X‐ray solution scattering
UV: ultraviolet

Protein structural rearrangements are fundamental to achieving biological function and enabling processes such as signal transduction, molecular transport, and enzymatic activity. These structural transitions range from subtle side‐chain rearrangements to large‐scale conformational changes, and often occur in a coordinated and sequential manner, reminiscent of mechanical components in an engine. The resulting structural states occur on a wide temporal scale, typically from picoseconds to milliseconds, and resolving these transient intermediates constitutes a major challenge in structural biology. Although high‐resolution techniques such as cryo‐electron microscopy (cryo‐EM) [1] and time‐resolved approaches for X‐ray crystallography [2] have greatly advanced our understanding of protein structure and dynamics, the methods cannot capture conformational transitions in solution at physiological temperatures, or involve rapid, highly flexible motions, or disordered regions. In such cases, time‐resolved X‐ray solution scattering (TR‐XSS) provides a complementary method capable of real‐time characterization of protein conformational dynamics under near‐native conditions [3, 4, 5, 6]. In light‐triggered TR‐XSS experiments, the protein reaction is initiated by a laser flash, either activating the protein directly as in the case of light‐sensitive proteins [7, 8, 9, 10, 11, 12, 13, 14, 15, 16, 17, 18, 19, 20], by photoinduced pH jump [21], or by releasing caged compounds (e.g., ATP) [22, 23, 24, 25], and structural changes are tracked by collecting X‐ray scattering profiles at defined time delays. Alternative triggers can be used, such as rapid mixing [26, 27], or temperature jumps [28, 29, 30], but are not within the scope of this protocol. Because changes in positions of all atoms in the solvated protein contribute to obtained difference scattering data, proteins can be studied in their native state without the need for crystallization, freezing, or chemical labeling. Light‐triggered TR‐XSS has been applied successfully to study highly diverse structural phenomena, such as protein folding [31, 32], dimerization [25], light‐induced processes [7, 8, 9, 10, 11, 12, 13, 14, 15, 16], temperature‐jump transitions [28, 29, 30, 33], but also structural rearrangements in small photoactive solutes [34] and solvents [35].

The recorded X‐ray scattering profiles provide one‐dimensional, spherically averaged intensity distributions from an ensemble of billions of molecules each with random orientation as a function of the momentum transfer. Therefore, TR‐XSS do not directly yield three‐dimensional structures, and the major methodological challenge lies within structural interpretation. Generation of candidate structures for structural refinement typically involves protein structures from cryo‐EM or X‐ray crystallography combined with homology modeling [24], AI‐driven modeling [36], or molecular dynamics (MD) simulations [22, 23, 24, 37], followed by comparing calculated theoretical scattering curves (from e.g., CRYSOL software [38]) to experimental difference profiles, although data‐driven refinement methods have also been proposed [39, 40].

In this protocol, a calcium‐transporting P‐type ATPase from *Listeria monocytogenes* (LMCA1) purified in n‐dodecyl‐β‐D‐maltoside (DDM) detergent is used as an illustrative example. The TR‐XSS data were collected at the dedicated time‐resolved beamline ID09 at the European Synchrotron Radiation Facility (ESRF) [24], where polychromatic X‐ray pulses are isolated and synchronized with a laser via an X‐ray chopper [41]. The processing and structural‐refinement workflow is also compatible with multipurpose small‐angle X‐ray scattering (SAXS) beam stations using fast detector readout [42, 43, 44]. It should be noted that the protocol details will depend upon protein target, so the presented workflow aims to constitute an adaptable framework.

## Materials

### TR‐XSS setup

1. Nanosecond laser system at UV wavelength, here; Ekspla NT 342‐10‐2H‐WW
2. Time‐resolved synchrotron beamline, here: ID09 at ESRF
3. Quartz capillaries (inner diameter = 0.4 mm)
4. Syringe or peristaltic pump with tubing inner diameter of 0.25 mm or 0.5 mm, respectively

### Computational setup

1. High‐performance computing resource access, here: High‐Performance Computing Center North (HPC2N)
2. MD simulation engine capable of targeted simulations, here: GROMACS v2019.4 [45] compiled with PLUMED v2.5.4 [46]
3. Homology modeling software, e.g. SWISS‐MODEL [47] or MODELLER [48]
4. Software for MD system setup; e.g. CHARMM‐GUI v3.7 [49, 50]
5. Software for calculation of SAXS patterns from protein structures, e.g. Crysol [38], FoXS [51], Pepsi‐SAXS [52], or WAXSiS [53]
6. Python with the txs package (https://gitlab.esrf.fr/levantin/txs), and our provided scripts: trxss_data_reduction.py, trxss_data_decomposition.py, trxss_crysol.py, trxss_structural_refinement.py, and trxss_pca_clustering.py

## Preparing the light‐triggered TR‐XSS experiment and data collection

### Light triggering

The laser wavelength should match the absorption of the light‐sensitive protein or the caged compound. Laser fluence must be sufficient to activate a substantial fraction of the protein molecules. Activation can be estimated from the absorbance characteristics and excitation probability/quantum yield, and can be optimized experimentally, for example, by time‐resolved Fourier‐transform infrared spectroscopy (FTIR) [54] or by titrating laser fluence and protein/caged compound concentrations at the synchrotron. For nitrophenylethyl (NPE)‐caged ATP, 1.5–10 mm has been used with inversely varied laser fluences of ~12–1 mJ·mm^−2^ [22, 25]. The optimal concentration‐fluence balance depends on sample limitations, that is, high caged‐compound concentration with low fluence allows reuse of precious protein samples across multiple cycles, whereas high fluence may be preferable when the protein is abundant but the caged compound is limiting. Note that excessive laser fluence can induce multiphoton absorption and local heating, which may deteriorate the protein signal or damage the sample.

In the LMCA1 experiment, NPE‐caged ATP was photolysed at 355 nm (ε = 430 M^−1^ cm^−1^). With 0.9 mJ·mm^−2^ fluence, 10 mm NPE‐caged ATP, and a quantum yield of 0.63 [55], we estimated that each laser pulse released ~1.7 mm ATP, in large excess of LMCA1.

### Timescale of interest

Determine the timescale of interest by spectroscopic measurements or biochemical activity assays, since this will influence the experimental setup. Fast dynamics in the nano‐ to microsecond range require a beamline equipped with a high‐speed chopper, whereas reactions occurring over tens to hundreds of milliseconds are better suited to beamlines with rapid detector readout. When using a caged compound, its photorelease should preferentially be faster than the protein dynamics being probed.

For LMCA1, the reaction cycle occurs on the order of tens of milliseconds, so scattering was recorded up to 200 milliseconds. NPE‐caged ATP releases ATP on the millisecond timescale [56], which is sufficiently fast to follow LMCA1 dynamics.

### Sample delivery

Choose the appropriate sample‐delivery method, for example, syringe or peristaltic pump, and define the required flow rate, temperature control, and sample‐to‐detector distance. The setup should cover both the small‐angle region (protein signal) and mid‐high‐*q* values up to ~2.4 Å^−1^ for solvent‐heating correction [3, 12]. Reversible reactions may be measured in sealed capillaries and allowed to relax between X‐ray pulses, which minimizes sample consumption [8]. For irreversible reactions, the flow rate must ensure that (i) removal of all activated sample before the next X‐ray pulse and (ii) activated sample remains in the probed area until X‐ray arrival. These limits depend on the repetition rate, laser‐spot dimensions, and the capillary inner diameter.

In the LMCA1 experiments, the sample was circulated through a 0.4‐mm quartz capillary using a peristaltic pump and returned to the reservoir. Because LMCA1 consumes the released ATP, the sample could be reused as long as sufficient NPE‐caged ATP remained, which enabled minimal sample quantities.

### Data collection scheme

Choose the repetition rate and acquisition mode according to the desired time delays to ensure that each X‐ray exposure probes a fresh sample volume. The X‐ray pulse length must be shorter than the shortest time delay to achieve the desired time resolution. Adjust the number of X‐ray pulses per image (integration time) to obtain sufficient signal. About 2‐ms total exposure time is typically adequate, with additional images collected until the desired signal‐to‐noise is reached.

For LMCA1, short time delays (< 25 ms) were collected at 10 Hz and long delays (> 25 ms) at 1 Hz. Because the repetition rate determined the minimum flow rate needed to clear the activated sample, we used 20 μL·min^−1^ at 10 Hz and 2 μL·min^−1^ at 1 Hz. The sample was probed with 20 μs, 18 keV X‐ray pulses, and scattering patterns were recorded on a Rayonix MX170‐HS detector positioned 380 mm from the sample with a helium cone to reduce air scattering (Fig. 1). For each time delay, 100 X‐ray pulses (i.e., total 2 ms) were integrated per image, and ~ 30 images were averaged. Positive delays were measured in randomized order and interleaved with negative (dark) controls to avoid systematic bias [51].

**Fig. 1:**
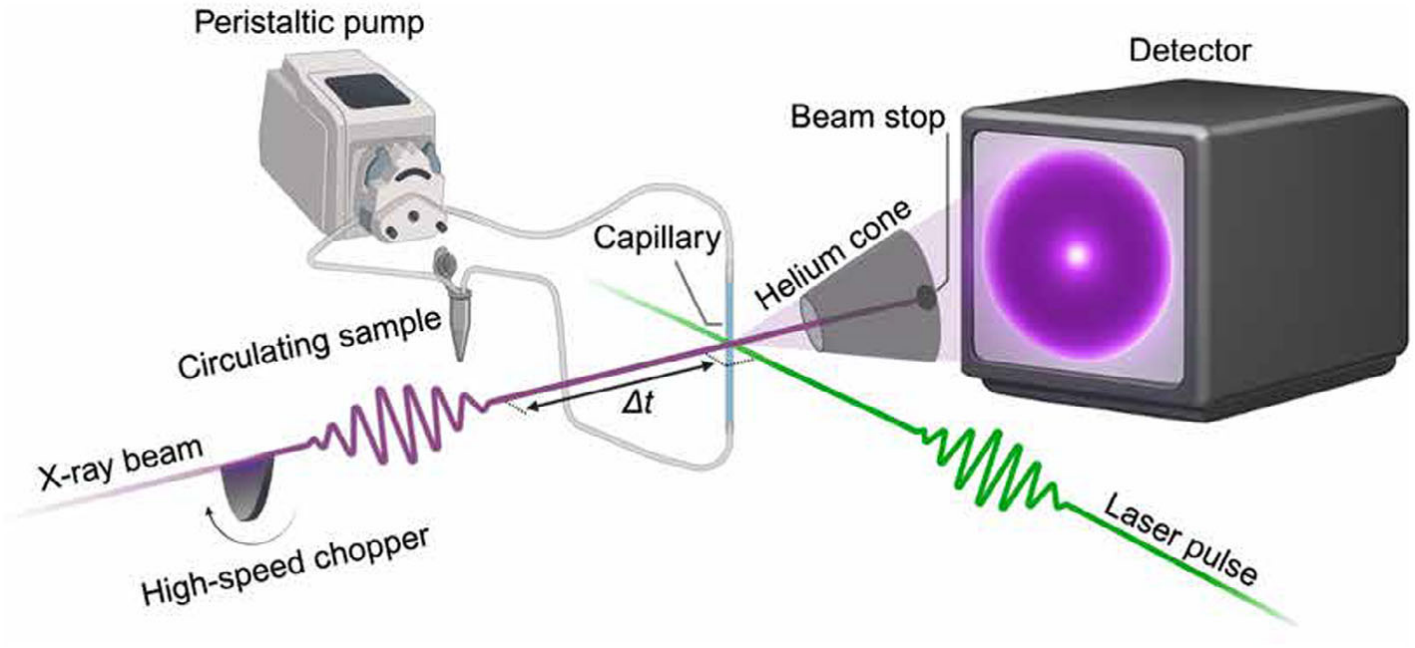
Schematic illustration of a light‐triggered time‐resolved X‐ray solution scattering (TR‐XSS) experimental setup. A peristaltic pump drives the sample through a closed loop into a capillary, where it is exposed to both an optical laser pulse (pump) and an X‐ray pulse (probe). The laser pulse (green) initiates the reaction and after a precisely controlled delay time (Δt), the X‐ray beam (purple) yields the structural information. A high‐speed chopper modulates the X‐ray pulses to synchronize with the laser excitation to ensure the desired temporal resolution. The beamstop blocks the unscattered X‐rays, while elastically scattered X‐rays pass through a helium cone onto the 2D detector.

## TR‐XSS data processing pipeline

In the following, we provide an overview of data processing steps. Data‐reduction procedures vary between beamlines depending on timing scheme, data format, and available software. The procedures and scripts provided here are compatible with ESRF ID09 data, and the txs Python package (https://gitlab.esrf.fr/levantin/txs) includes functions for these processing steps. Note that kinetic modeling will depend on the protein reaction mechanism and the associated script should be considered as a framework that can be adapted accordingly.

### Generation of absolute X‐ray scattering curves, S(q)

The 2D detector images are azimuthally integrated to obtain 1D scattering profiles S(q) as a function of the momentum‐transfer q (Fig. 2A,B). The details of the integration depend on the experimental setup, including X‐ray beam energy, sample‐to‐detector distance, detector type, pixel binning, and beam‐center calibration.

**Fig. 2:**
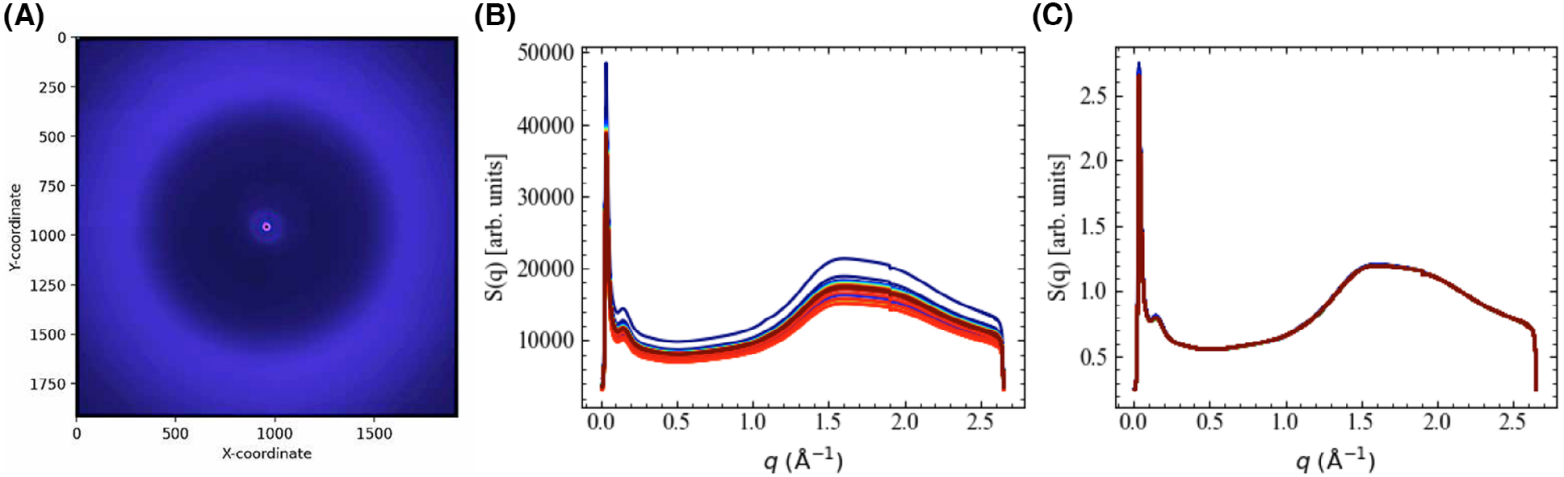
Azimuthal integration and normalization of TR‐XSS data. (A) 2D detector image with concentric rings of isotropic scattering. (B) Absolute X‐ray scattering curves obtained after azimuthal integration across the different time delays. (C) The corresponding curves normalized over a *q*‐range (2.1–2.2 Å^−1^).

### Preparation before generating difference X‐ray scattering curves

Laser‐induced structural changes produce only small variations relative to the total scattering intensity, and even minor fluctuations in overall amplitude, such as those resulting from drift in beam intensity, can obscure the true signal. To correct for this, each 1D scattering curve is scaled by normalizing its intensity around an isosbestic point, where no laser‐induced scattering change is expected (Fig. 2C). For water, commonly used isobestic regions are centered at ~1.5 Å^−1^ [3] or ~ 2.1 Å^−1^ [12]. Normalization is typically performed over a narrow range of ±0.05 Å^−1^ around the chosen point.

### Generation of difference X‐ray scattering curves, ΔS(q)

Laser‐off reference curves are subtracted from the laser‐on curves to yield difference scattering curves in which background contributions from solvent scattering, inactive protein molecules, experimental drift, and radiation damage are largely canceled out (Fig. 3). For time‐resolved beamlines that acquire images corresponding to individual time delays, the generation of difference X‐ray scattering curves is usually done by weighting and then subtracting two flanking laser‐off curves. At multipurpose beamlines using fast detector readout where full time‐delay series are collected, the same principle is applied to entire image blocks, that is, the preceding and subsequent laser‐off series are weighted and subtracted timepoint‐by‐timepoint across the laser‐on series [57].

**Fig. 3:**
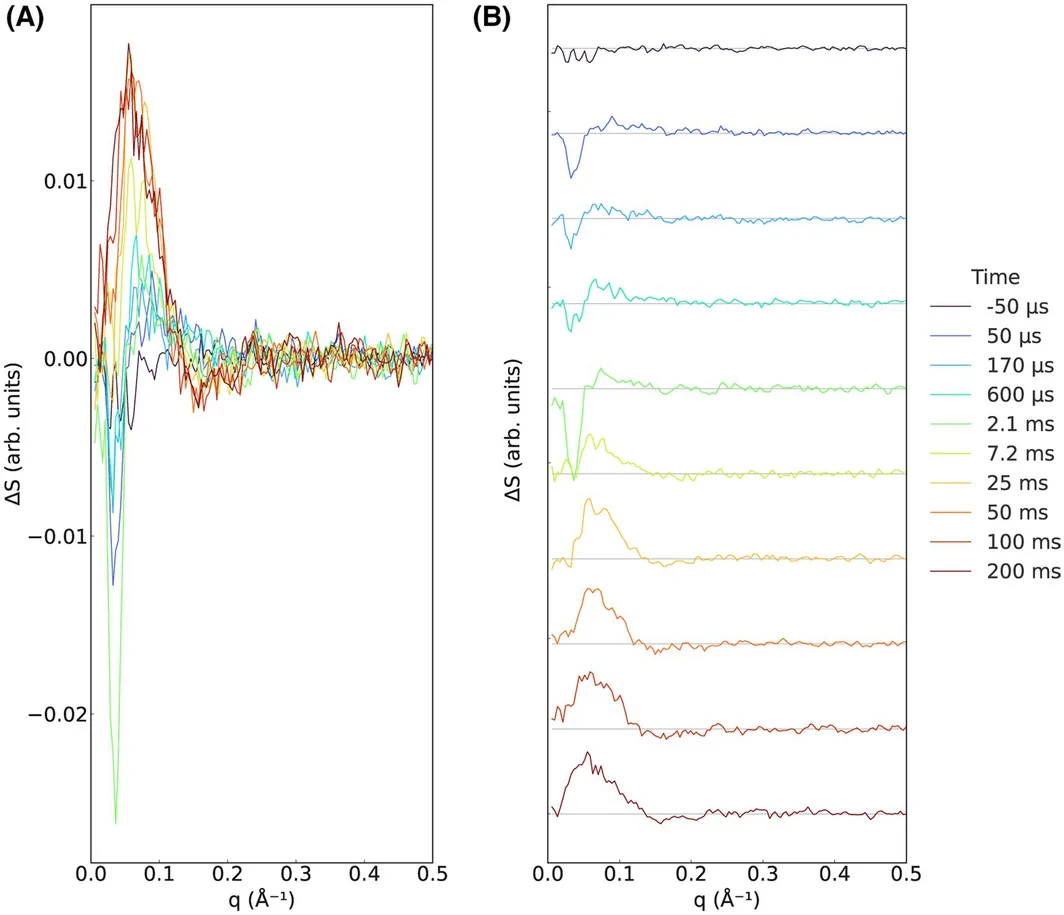
Difference X‐ray scattering curves. (A) Difference X‐ray scattering profiles, ΔS(q), as a function of the scattering vector q for each time delay, calculated by subtracting two flanking reference time points at −50 μs from each time delay. (B) Same data shown with vertical offsets for clarity. Time points range from 50 μs to 200 ms, color‐coded from blue to red. The data were merged from two acquisition modes (10 Hz and 1 Hz).

### Outlier removal

After calculating difference scattering curves, outlier traces must be identified and removed. In our workflow, the txs package applies a reduced chi‐squared (χ^2^) threshold (default: 95th percentile). Several alternative approaches have been reported, including rejection of difference data deviating by >3 standard deviations from the mean [22, 37], removal of absolute scattering curves that fall outside the median range in a high‐q window 2–2.5 Å^−1^ [37], or SVD‐based rejection of curves with anomalous contribution to the first left singular vector [33]. For well‐behaved datasets, these methods typically yield similar outlier fractions (~5–15%). Datasets affected by bubbles, aggregates, capillary deposits, or intermittent beam loss may require additional manual inspection, since large clusters of affected images can bias automated criteria. In such cases, plotting an integrated intensity over a defined *q*‐range is an effective way to identify and discard problematic segments before applying the automated outlier rejection.

For the LMCA1 dataset, difference curves that deviated by more than 3.5 standard deviations were removed, which corresponded to 22% of the data.

### Averaging of individual time delays

For each time delay, the accepted difference scattering curves are combined by computing their mean and standard deviation to yield averaged ΔS(q) curves and associated uncertainties.

### Merging of datasets

Datasets collected at different acquisition modes (e.g., 1 Hz and 10 Hz) can be merged to extend time coverage. A shared time point is required for scaling. Once aligned, the combined dataset is subjected again to outlier removal and averaging.

For LMCA1, data collected at 10 Hz (< 25 ms) and 1 Hz (> 25 ms) were scaled using the common 25 ms time delay and subsequently merged.

### Heating correction

Absorption of the triggering laser leads to rapid solvent heating, which contributes to a characteristic signal in the difference scattering curves. To remove this contribution, a heating‐only difference curve is scaled to the protein data over the temperature‐sensitive q‐range (1.5–2.2 Å^−1^) and subtracted, leaving only structural changes arising from the photoreaction (Fig. 4) [58]. Heating references can be obtained in several ways. A convenient approach is to measure scattering from a fluorescent dye that absorbs at the laser wavelength and converts the energy into heat, which produces a strong and reproducible reference using the same laser conditions as in the experiment. Alternatively, buffer containing only the caged compound or time points in which the protein has fully relaxed can be used [3, 8]. The heating response can also be recorded on a protein sample with an infra‐red (IR) laser that heats the solvent without triggering any photoreaction [37].

**Fig. 4:**
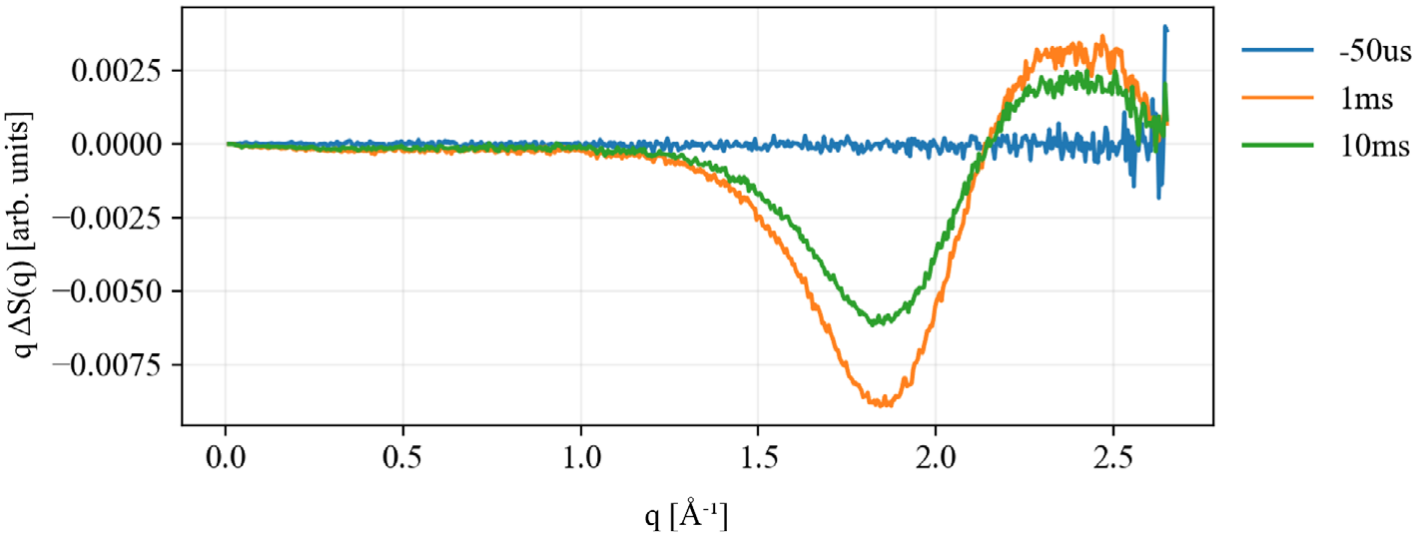
Reference curves used for heating correction. Difference scattering curves at 1 ms and 10 ms time delays obtained from a fluorescent dye to generate a heating curve.

For LMCA1, the heating reference was obtained using the fluorescent dye Acid Yellow 9 (CAS 74543‐21‐8), which absorbs light between 200 nm and 550 nm at pH 6.8 [59] and was excited at the same wavelength (355 nm) used for caged ATP photorelease.

## Data decomposition and kinetic modeling

The difference scattering curves form a time series in which each time delay reflects the combined signal of all structural species present at that moment. For interpretation, however, it is the species‐specific difference curves and their associated time‐dependent populations that are of primary interest. Recovering these underlying components requires data‐decomposition approaches, which may vary depending on the system. The methods outlined here are intended as general guidelines and additional system‐specific information may be necessary in some cases.

### Singular value decomposition (SVD) analysis

SVD is a useful first step in TR‐XSS data decomposition to provide an overview of the number and character of time‐dependent structural signals. The SVD analysis expresses the dataset as time‐independent scattering curves (left singular vectors, LSVs), time‐dependent coefficients (right singular vectors, RSVs), and corresponding singular values [60, 61, 62]. While SVD components do not directly correspond to physical species, they are valuable for guiding model selection. The number of significant components can be estimated from the magnitude of the singular values and from the autocorrelation [12, 63]. Visual inspection of the LSVs and RSVs can help select structured vectors that likely represent real signals, rather than noise and rapid fluctuations. Approximate time constants can be obtained by fitting sums of exponentials to the RSVs using shared time constants [64]. Together, these estimates reduce the number of kinetic models to consider.

For LMCA1, SVD indicated two dominant components from large singular values and highly correlated LSVs and RSVs [24]. The first two LSVs showed clear structural features, whereas higher components appeared noise‐like (Fig. 5).

**Fig. 5:**
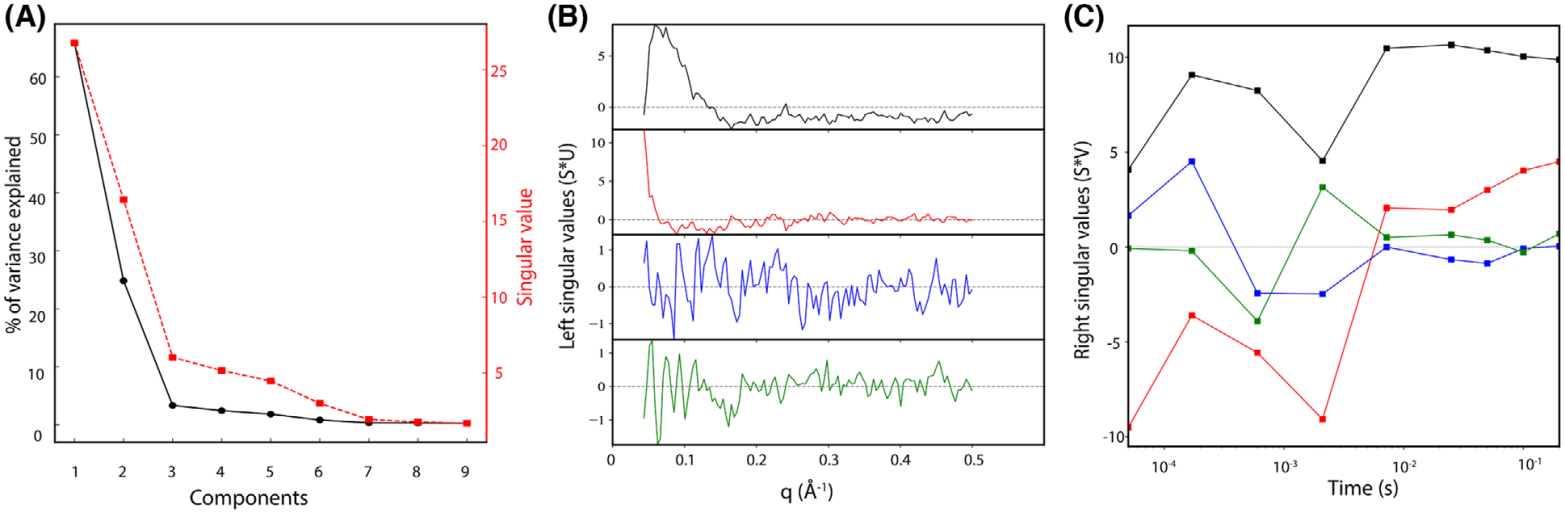
Singular Value Decomposition (SVD) analysis of LMCA1 TR‐XSS data. (A) The percentage of variance explained and singular value by each component, (B) Left singular vectors corresponding to the first four components explaining 96% of the total variance, and (C) The associated right singular vectors. Reproduced from Prabudiansyah *et al*., 2024, licensed under CC BY‐NC [24].

### Kinetic modeling

To obtain physically meaningful time‐independent scattering curves, the TR‐XSS data must be decomposed using a kinetic model that describes how the populations of the underlying structural components evolve over time. The choice of model strongly influences the interpretation and should be guided by SVD analysis, prior knowledge of the reaction, and the overall signal‐to‐noise of the dataset. High‐quality data can support more complex models, whereas weaker datasets typically require simpler descriptions. In many TR‐XSS studies, simple exponential models, including sequential or parallel first‐order kinetics, have provided satisfactory fits, for example in rhodopsins [8, 37], P‐type ATPases [22, 24], and phytochromes [13]. When such models do not capture the observed dynamics, more flexible models can be tested, such as parallel pathways modeled by stretched or bifurcated exponential models [32, 65], branched pathways [29], or system‐specific mechanistic models [63].

Model evaluation is commonly achieved by reconstructing the scattering at each time point from the fitted basis spectra and comparing the resulting time‐dependent weights to the model predictions. Large deviations, such as transient rise and then fall or a lasting plateau, suggest that the model does not capture the data. Over‐parameterized models may produce basis spectra that are not independent or that partially fit noise and should be interpreted with caution.

For LMCA1, the data were well‐described by a sequential two‐state model that resulted in early and late state basis spectra that captured the structural evolution of the transport cycle (Fig. 6).

**Fig. 6:**
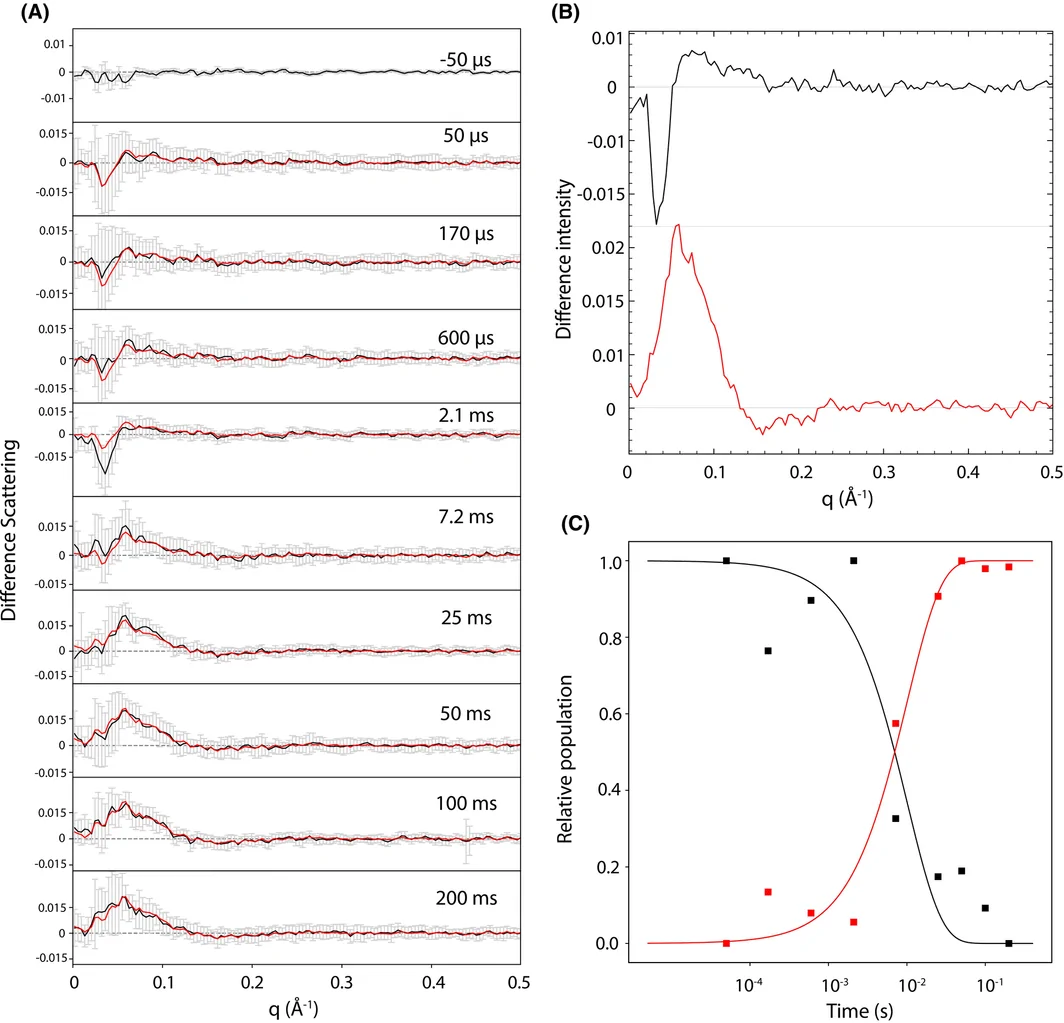
TR‐XSS analysis of the LMCA1 transport cycle. (A) Experimental difference scattering curves (black lines) with standard deviations (gray bars), overlaid with reconstructed curves from a two‐state kinetic model (red lines). (B) Time‐independent basis spectra from TR‐XSS modeling, showing early (black) and late (red) states for the LMCA1 transport reaction. (C) Time evolution of the relative population of the two kinetic states across the experimental time points. Reproduced from Prabudiansyah *et al*., 2024, licensed under CC BY‐NC [24].

## Structural interpretation of the TR‐XSS data

Because TR‐XSS reports an ensemble‐averaged, low‐resolution signal, high‐resolution structures cannot be accessed. Instead, the experimental difference curves are interpreted by comparison with theoretical difference scattering calculated from candidate structures.

### Generation of candidate structures

A range of approaches can be used to generate structural models for comparison with TR‐XSS data:

- Molecular dynamics (MD) simulation: MD simulations are widely used to explore conformational space [66], either by equilibrium sampling or enhanced‐sampling techniques such as targeted MD [67], metadynamics, or accelerated MD, which can overcome free energy barriers to explore rarely sampled states [68, 69, 70, 71]. This approach is described in more detail below.
- Manual structural manipulation: Intermediate conformations can be generated by manual adjustment of known structures (e.g., domain rotations, loop repositioning) [8], followed by energy minimization or short restrained MD to ensure structural integrity.
- Normal Mode Analysis (NMA): NMA provides estimates of large‐scale collective motions at low computational cost and can efficiently generate plausible protein conformations using elastic‐network models [72].
- AI‐based structure prediction: Methods such as Alphafold2 [73] and ColabFold [74] can generate additional candidate models, either as starting points for MD simulations or as a direct source of conformational sampling. Techniques that modify or subsample the input multiple sequence alignment (MSA) [75, 76, 77] have been shown to produce alternative conformations relevant to functional transitions [78, 79].

### Targeted MD setup for generating transition state conformations

- Selection of starting and target structures: Identify a set of experimentally determined or predicted protein structures as starting and target conformations. Homology models can be created with SWISS‐MODEL [80] or Modeller [81].
- System building: Systems can be prepared using automated platforms such as CHARMM‐GUI [49, 50], which builds protein systems in solution or membrane environments using standard force fields. Alternatively, systems can be constructed manually in packages such as GROMACS [45], AMBER [82], or NAMD [83] by selecting an appropriate force field (e.g., CHARMM36 [84], AMBER99SB [85], OPLS [86]), defining the simulation box, solvating with explicit water, and adding counterions [87].
- Equilibration: After energy minimization to remove steric clashes, the system is equilibrated with gradually released position restraints to allow relaxation before applying targeted dynamics.
- Targeted MD production runs: Targeted MD is then used to drive the system between the chosen starting and target conformations while sampling intermediate states. A smoothly increasing force constant is commonly applied to balance adequate sampling with efficient progression toward the target structure. In GROMACS [45] combined with PLUMED [46], this can be implemented using an RMSD collective variable with a force constant that ramps up during the simulation. Convergence and sampling should be evaluated to ensure that the conformational space has been sufficiently sampled by, for example, projecting the trajectory onto relevant collective variables, such as distances or angles.
- Extraction of candidate structures: Representative structures are extracted at regular intervals for theoretical scattering calculations.

For LMCA1, homology models based on SERCA structures [88] were generated using SWISS‐MODEL 3.3.0 [47, 80] and inserted into a POPG lipid bilayer (264 lipids) using CHARMM‐GUI 3.7 [49, 50]. Systems were solvated with TIP3 water and 150 mM KCl, and simulations were run with GROMACS 2019.4 [45] and PLUMED 2.5.4 [46]. After energy minimization (steepest descent, F_max < 1000), equilibration was conducted in six steps (three × 125 ps at 1 fs, three × 500 ps at 2 fs). Targeted MD simulations of 30 ns were performed using an RMSD collective variable with a force constant ramped from 0 to 3000 kJ·mol^−1^·nm^−2^. From these trajectories, 3612 structures (one every 100 ps) were selected for theoretical scattering analysis.

### Calculation of theoretical scattering curves

- Choose the software: CRYSOL [38], WAXSiS [53], FoXS [51], or Pepsi‐SAXS [52].

CRYSOL settings: Increasing the number of harmonics may improve the high‐q fit but increases computation time. The maximum *q*‐value (*q*max) should cover the experimental *q*‐range to ensure proper comparison. Hydration layer parameters, including density and thickness, are important for accurately modeling solvent effects. For output options, *solution scattering* is recommended for most protein studies. *In vacuo* and *solvent/border* modes may be used for specialized applications [22, 37].

For LMCA1, CRYSOL 2.8.4 [38] was used to calculate theoretical scattering curves for all candidate structures using a scattering angle < 0.6 Å^−1^, up to 50 harmonics, and a 17‐order Fibonacci grid.

### Comparison of theoretical and experimental difference scattering curves

Calculated X‐ray scattering curves are used to generate theoretical difference scattering curves, which are then compared with the experimental TR‐XSS data using metrics such as R‐factor or chi‐squared values. A major challenge is that many ground/excited structure pairs can fit the data equally well. This ambiguity is most prominent when both ground and excited states are fitted simultaneously. Constraining the ground state greatly narrows the conformational search space and facilitates interpretation.

- Defined ground state: When a reliable ground state structure (or a small set of related structures) is available, the conformational search is restricted and lowers the risk of selecting outliers. Short relaxation simulations may mitigate crystal‐packing artifacts.
- Using static SAXS: Ground‐state SAXS data can provide candidate structures or ensembles to serve as input for TR‐XSS refinement.
- Undefined ground state: If the ground state is uncertain, multiple candidate structures must be tested against all excited‐state structures. This typically yields several well‐fitting pairs, which should be evaluated collectively rather than relying on a single best fit.
- Ensemble descriptions: In some systems, the ground or excited state is best represented as an ensemble. In such cases, ensemble‐optimization methods can aid refinement [31, 32, 88].

For LMCA1, theoretical scattering curves were computed for all candidate structures in both states, the theoretical difference curves were scaled to the experimental data, and R‐factors were used to rank fits.

### Clustering and visualization

Clustering and visualization help interpret the refined structures. Structural similarity can be quantified using RMSD, protein‐specific metrics (e.g., distances or angles), or reduced‐dimension representations such as principal components from PCA [89].

- Clustering:
  ○ If the ground state is a single structure, clustering is applied to the excited states.
  ○ If multiple ground states are used, pairs should be clustered using a pair‐distance metric (distance between ground structures + distance between excited structures).
- Visualization:
  ○ Direct molecular visualization becomes difficult when pairs are heterogeneous or the conformational change is subtle. Reduced‐dimensional plots, e.g. PCA or scatter plots of distance/angle metrics, provide clearer views of the diversity of well‐fitting pairs (Fig. 7).
- Interpret diverse solutions: When many structurally distinct pairs fit well, further filtering is needed. Consider:
  ○ Consistency with prior knowledge: do any clusters align with expected ground/excited states?
  ○ Magnitude of structural change: Ground and excited structures that are too similar often arise from overfitting enabled by free scaling and typically do not form meaningful clusters.
  ○ Trends across pairs: If most pairs show a similar motion (e.g., closing movement), that motion is likely real. Ground‐state refinement with static SAXS may reduce excited‐state variability. In some cases, ensemble descriptions may be more appropriate.
  ○ Multiple excited states or basis patterns: Solutions should share similar ground states since all excited states arise from the same ground state.
  ○ Scaling factors: Solutions from different basis patterns should show comparable scale factors because they derive from the same dataset.

**Fig. 7:**
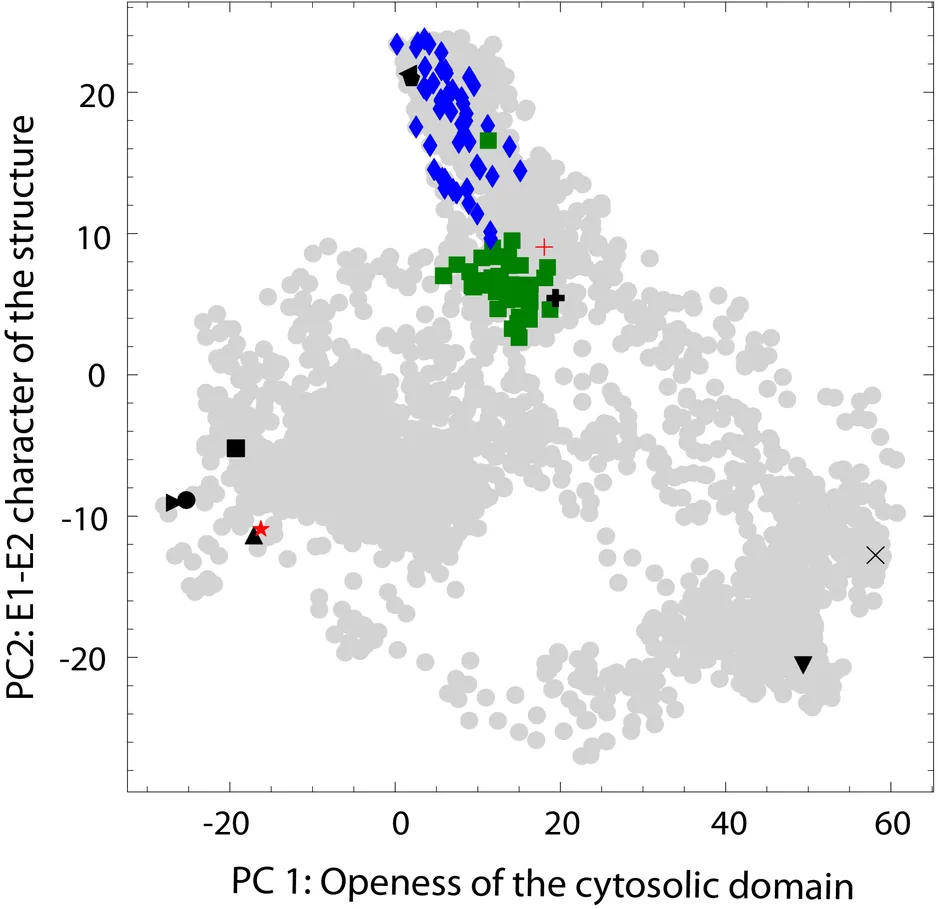
Projection of homology models (black symbols) and simulated structures (gray circles) onto principal components 1 and 2. The Ca^2+^‐bound E1 (inward‐facing) and E2P (outward‐facing) states are indicated by a red plus sign and red star sign, respectively. The 100 best‐fitting pre‐pulse structures are shown as green squares and the corresponding excited‐state conformations as blue diamonds. Reproduced from Prabudiansyah et al., 2024, licensed under CC BY‐NC [24].

In the LMCA1 example [24], the 100 best‐fitting pairs (lowest R‐factors) were clustered using agglomerative hierarchical clustering (distance threshold 10) to identify ground‐ and excited‐state ensembles. Because LMCA1 undergoes combined domain translation and rotation, the pairs were projected onto the first two PCA components of the homology models (Fig. 7). The plot reveals a single dominant transition, corresponding to closure of the cytosolic headpiece, consistent with the phosphorylation step of the P‐type ATPase reaction cycle.

## Tips and tricks

1. High sample purity and protein concentration are critical. Any aggregation, precipitation, or degradation will introduce scattering artifacts. Too low protein concentration will reduce the signal‐to‐noise ratio.
2. Capillaries should be uniform in diameter and wall thickness to avoid uneven illumination.
3. Rinse the flow loop well with water several times to remove leftover detergent or protein that could affect the scattering results.
4. Heat can hide small changes. Always run a buffer or dye‐only control and create heat correction curves for key time points.
5. Always verify that kinetic modeling assumptions (e.g., number of states, irreversibility) match the biological context. Overfitting can hide the real mechanism.
6. Large portions of recorded images may be unusable due to bubbles, aggregates, debris, or X‐ray beam loss. A few bad images can usually be removed through standard outlier rejection, but if many are affected, they must be filtered manually or using a defined metric, such as the integrated intensity over a chosen q‐range, to separate the good data from the bad data.

## Conflict of interest

The authors declare no conflict of interest.

## Author contributions

F.S. was involved in conceptualization, methodology, investigation, data curation, writing‐original draft, writing – review and editing, and visualization. F.O. was involved in conceptualization, methodology, investigation, validation, data curation, and writing – review and editing. K.M. was involved in conceptualization, methodology, validation, and writing – review and editing. M.A. was involved in conceptualization, methodology, validation, supervision, writing – review and editing, and funding acquisition.

## Data Availability

All analysis scripts used in this study are available at Zenodo: https://doi.org/10.5281/zenodo.17061584.
